# Treatment of Oral Candidiasis Using Photodithazine^®^- Mediated Photodynamic Therapy *In Vivo*

**DOI:** 10.1371/journal.pone.0156947

**Published:** 2016-06-02

**Authors:** Juliana Cabrini Carmello, Fernanda Alves, Fernanda G. Basso, Carlos Alberto de Souza Costa, Vanderlei Salvador Bagnato, Ewerton Garcia de Oliveira Mima, Ana Cláudia Pavarina

**Affiliations:** 1 Department of Dental Materials and Prosthodontics, Araraquara Dental School, UNESP- Univ Estadual Paulista, Araraquara, SP, Brazil; 2 Department of Physiology and Pathology, Araraquara Dental School, UNESP- Univ Estadual Paulista, Araraquara, SP, Brazil; 3 Physics Institute of São Carlos, USP–University of São Paulo, São Carlos, SP, Brazil; Massachusetts General Hospital, UNITED STATES

## Abstract

This study evaluated the effectiveness of antimicrobial photodynamic therapy (aPDT) in the treatment of oral candidiasis in a murine model using Photodithazine^®^ (PDZ). This model of oral candidiasis was developed to allow the monitoring of the infection and the establishment of the aPDT treatment. Six-week-old female mice were immunosuppressed and inoculated with *C*. *albicans* to induce oral candidiasis. PDZ-mediated aPDT and nystatin treatment were carried out for 5 consecutive days with one application per day. The macroscopic evaluation of oral lesions was performed. After each treatment, the tongue was swabbed to recover *C*. *albicans* cells. Viable colonies were quantified and the number of CFU/ml determined. The animals were sacrificed 24 hours and 7 days after treatment and the tongues were surgically removed for histological analysis and analysis of inflammatory cytokines expression (IL-1, TNF-α and IL-6) by RT-qPCR. Data were analyzed by two-way ANOVA. PDZ-mediated aPDT was as effective as Nystatin (NYS group) in the inactivation of *C*. *albicans*, reducing 3 and 3.2 logs_10_ respectively, 24 h after treatment (p<0.05). Animals underwent PDZ-mediated aPDT showed complete remission of oral lesions, while animals treated with NYS presented partial remission of oral lesions in both periods assessed. Histological evaluation revealed mild inflammatory infiltrate in the groups treated with aPDT and NYS in both periods assessed. The aPDT induced the TNF-α expression when compared with the control (P-L-) (p<0.05), 24 h and 7 days after treatment. In summary, the murine model developed here was able to mimic the infection and PDZ-mediated aPDT was effective to treat mice with oral candidiasis.

## Introduction

*Candida* species are part of the normal oral microbiota [1] being *Candida albicans* the most common species associated with clinical manifestations, which range from superficial mucocutaneous lesions, such as oropharyngeal candidiasis (OPC), to disseminated forms of the infection [2]. OPC is the result of adhesion and penetration of fungal species into the oral tissues [3] and it has a high incidence in patients that use immunosuppressive drugs, broad-spectrum antibiotics, anti-diabetic mediations, anticancer therapies, and in patients with the acquired immunodeficiency syndrome (AIDS) [4].

The use of topical or systemic antifungal agents (azole, polyenes) for the treatment of OPC has resulted in the development of resistant *Candida* species [5]. Additionally, the organization of microorganisms in biofilms is a protective shell, enabling the survival of these pathogens even in unfavorable conditions and providing high resistance to antifungal agents [6]. Considering the increased incidence of resistant pathogens to conventional antifungal treatments and drug toxicity, studies have searched for strategies to control fungal species.

Photodynamic therapy is a common anticancer treatment that has been indicated for the inactivation of microorganisms (antimicrobial Photodynamic Therapy—aPDT) [7–9]. The mechanism of action of aPDT requires the use of a photosensitizing agent (PS), and the application of light corresponding to the absorption band of PS [8]. The interaction of the appropriate wavelength of light with the PS in the presence of oxygen results in reactive oxygen species (ROS) capable of inducing death of microorganisms by oxidative damage [10].

The PS drugs used in the antimicrobial aPDT are predominantly from the families of phenothiazinium dyes [7,8], porphyrins [11], 5-aminolevulinic acid (ALA) [12] and phenothiazines [13]. The use of distinct PSs have shown effects on viability of *C*. *albicans*, for example, methylene blue (450 and 500 mg/L) associated with laser light promoted 3 log_10_ reduction in the *C*. *albicans* viability [14], while Photogem^®^ (500 and 1000 mg/L) associated with LED was able to reduce 1.59 log_10_ in the viability of *C*. *albicans* [15]. In these studies the reduction in the number of microorganisms was only achieved when aPDT was applied together with high concentrations of PS.

Photodithazine^®^ (PDZ), a second-generation photosensitizer, is a glucosamine salt of chlorin (e_6_) derivative soluble in water [16]. This PS is characterized by shorter periods of photosensitization, longer activation wavelengths and higher yields of singlet oxygen than first-generation PS [17]. The molecules of PDZ are able to penetrate in the biological membrane, which is expected to improve PDT action [18]. *In vitro* studies have shown that PDZ associated with visible light was effective in the inactivation of cell suspensions of *C*. *albicans* and *Candida guilliermondi* [19–20], and single [21] and mixed biofilms of *Candida* spp [22]. Moreover, an in vivo study showed that 100 mg/L of PDZ associated with LED light reduced 4.36 log_10_ of the cell viability of *C*. *albicans* on the tongue of mice with induced oral candidiasis [23]. However, only one application of aPDT was made and the follow-up of the macroscopic evolution of *Candida* lesion was not performed.

Thereby, given the prevalence of OPC and the clinical similarity of this infection between mice and human host [24], it is essential to carry out *in vivo* studies that present infections caused by fungal biofilms, so that conditions close to the clinical situations can be simulated and evaluated. In a murine model of oral candidiasis used in previous studies [15, 23, 25–26], the lesions on the dorsum of the tongue were maintained for up to 7 days and the effectiveness of the antifungal treatment was assessed 24 hours after treatment administration. For this reason, in the present investigation, it was used an animal model of oral candidiasis in which induced infection was maintained over a longer period of time. Thus, it was possible to assess the effectiveness of successive applications of PDZ-mediated aPDT, simulating a possible clinical treatment for this infection. In addition, to understand the effect of aPDT on the host cells, it was evaluated the gene expression ratio of pro- and anti-inflammatory cytokines after treatments.

## Materials and Methods

### Photosensitizer and light source

The photosensitizer used in this study was PDZ, a chlorin e_6_ derivative (VETAGRAND, Co, Russia). Stock solutions of PDZ diluted in carboxiethilcellulose hydrogel (Pharmacy Santa Paula, Araraquara, SP, Brazil) at a concentration of 100 mg/L were prepared, storage at room temperature and kept in the dark until use.

The red LED light device had band I absorption maxima at longer wavelengths (λmax) 650–670 nm [16] (LXHL-PR09, LUXEON^®^ III Emitter, Lumileds Lighting, San Jose, California, USA) and was designed by the Physics Institute of São Carlos, USP, (University of São Paulo, São Carlos, SP, Brazil). The LED power density was 22.3 mW at 660 nm.

### Ethics Statement

The study was conducted according to the national (CONCEA—National Association for Animals Experiments Control: http://concea.mct.gov.br) and institutional laws and it was approved by the Ethics Committee on Animal Use (CEUA), of the Araraquara Dental School, Sao Paulo State University, UNESP (Authorization number 24/2012). All surgery was performed under ketamine anesthesia, and all efforts were made to minimize suffering.

### Animals and experimental oral candidiasis

A total of 126 6-week-old female Swiss mice specific pathogen free [SPF], with 20g of weight approximately, were used for all animal experiments. The animals were allocated to experimental groups by randomization before each experiment. Thus, they were kept in cages housing 5 animals in a temperature-controlled room (23 ± 2°C). Standard chow and water were given *ad libitum*.

The reference strain *C*. *albicans* ATCC 90028 (Rockville, MD) was used to induce oral candidiasis in mice. It was thawed and reactivated in Sabureaud Dextrose Agar medium culture (SDA) at 37°C for 48 hours. Then the strain was cultured in tubes with 5 mL of RPMI 1640 at 37°C for 24 hours. The tubes were centrifuged at 18.000 x*ɡ* for 10 minutes and the supernatant discarded. The cells were centrifuged at 18.000 x*ɡ* for 10 minutes, rinsed with sterile saline and resuspended in 2 ml of sterile saline (10^7^ CFU/ml).

To induce candidiasis in the animals, we used the method previously described by Takakura et al. (2003) [25], reproduced by our group [19–20], with some modifications. For this purpose, the animals were immunosuppressed with subcutaneous injections of prednisolone on days 1, 5, 9 and 13 of the experiment at a dose of 100 mg/kg body weight according to the period evaluated (24 hours and 7 days after treatment). Tetracycline (0.83 mg/mL) was administered in the drinking water of animals throughout the trial period. On day 2, the animals were sedated with chlorpromazine hydrochloride 0.1 ml (2 mg/mL) and mini-sterile swabs were soaked in *C*. *albicans* suspension and rubbed on the dorsum of the tongue for 30 seconds to produce oral candidiasis.

### Treatments and microbiological evaluation

**Fig 1:**
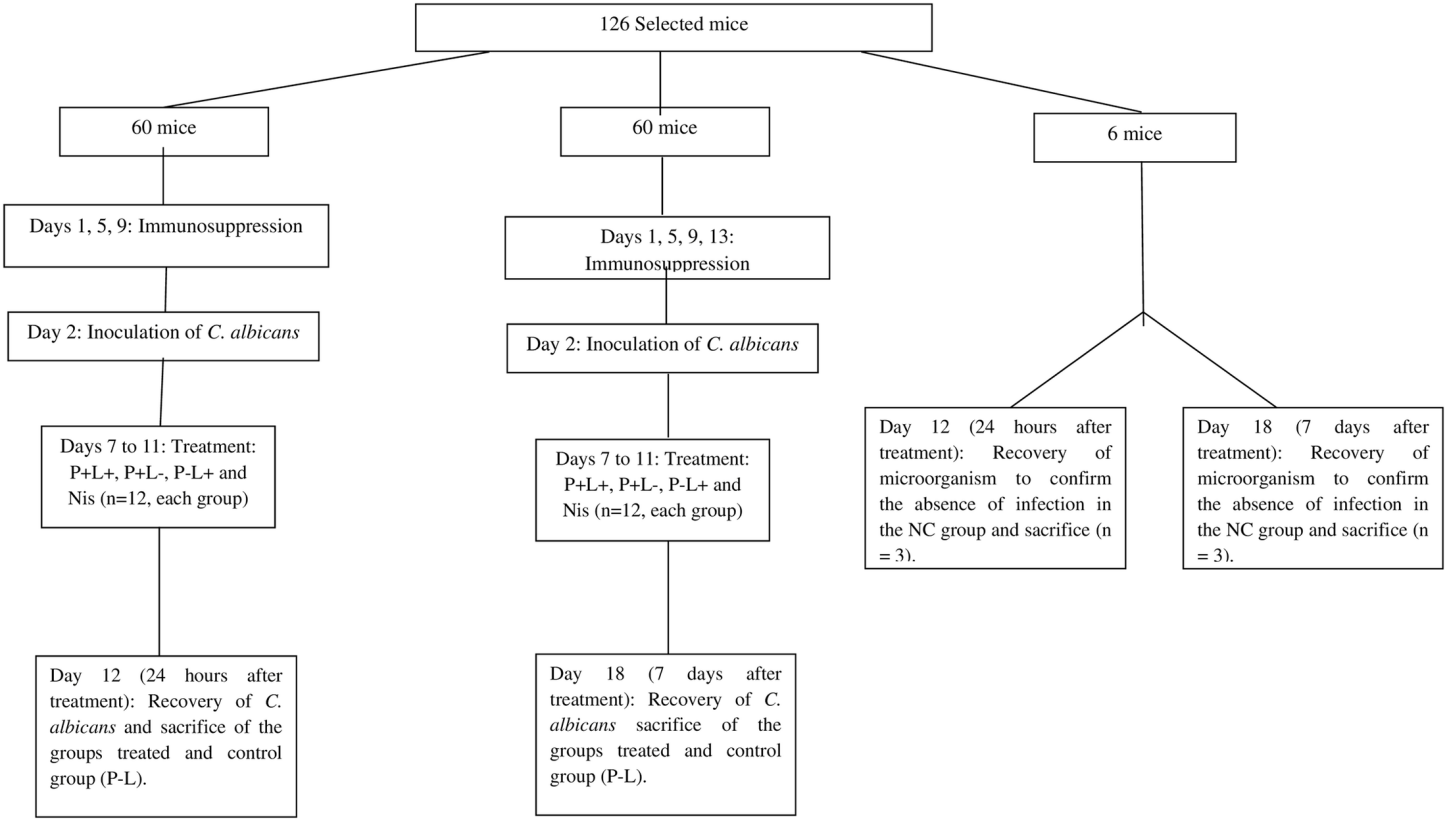
Study design: Tetracycline hydrochloride was given in drinking water during days 1 to 18; the P+L+ group corresponded to the animals treated with PDZ and LED light (P+L+); the P-L+ group corresponded to evaluation of LED light alone; the P+L- group corresponded to evaluation of the PS alone; the P-L- group corresponded to infected animals that did not receive any treatment; the NC group corresponded to the healthy and untreated animals.

On day 7, the infection was presented as verified by visual inspection, the animals were randomly divided into groups and treatments were performed daily from day 7 to day 11 (Fig 1 Study design). For this purpose, the animals were anesthetized with an intraperitoneal injection of ketamine at a concentration of 100 mg/kg body weight (National Pharmaceutical Chemistry Union S/A, Embu, SP, Brazil) and xylazine at 10 mg/kg body weight (Veterinary JA Ltda., Sponsor Paulista, SP, Brazil). The tongues of the animals were gently taken out of the oral cavity and PDZ diluted in hydrogel (100 mg/L) was applied, as previously described [23]. The animals remained in the dark for 20 minutes (pre-incubation time). After this period, the tongue of the mice was illuminated with LED light for 14 minutes, resulting in total fluency equivalent to 37.5 J/cm^2^ (P+L+ group) [23]. The effect of the isolated application of PS (P+L-) and the LED light (P-L+) was also evaluated. After all treatment the PS was not removed from the oral cavity. The untreated control group (P-L-) did not receive any PS or light. The negative control group (NC) did not receive *C*. *albicans* inoculation or any treatment. Additionally, there was a positive control group in which animals were treatment with topical antifungal nystatin oral suspension (NYS) at 100,000 IU. The drug was applied on the dorsum of the tongues of the anesthetized animals, once a day for 5 days. The treatments were applied daily and the microbiological evaluation was performed 24 hours and 7 days after 5 applications of the treatments by swabbing. It was performed to avoid the decrease of colony forming units (CFU/mL) during the treatment, because the successive swabbing of the oral lesions could interfere in the final results of the treatments by reducing yeast load in the local tissue. Twelve animals were used for each experimental condition, except in the group NC (n = 3).

At 24 hours and 7 days after 5 consecutive days of treatment, *C*. *albicans* cells were recovered from the tongue of mice. For this, the tongues were swabbed for 1 minute. The swabs were transferred to tubes containing 1 ml of saline solution and vortexed during 1 minute to detach *C*. *albicans* cells. Then, serial dilutions were made from the samples contained in the tubes, and plated in duplicate onto SDA culture medium with 5 μg/ml of chloramphenicol. After 48 hours of incubation at 37°C, the viable colonies were counted and the values of CFU/ml were determined.

### Macroscopic analysis of candidiasis lesions

The lesions on the tongue of the animals were also assessed by standardized photographs taken before the beginning of the proposed treatments, and after 24 hours and 7 days of the last application. All the photographs were taken with the same digital camera (Sony Cyber-Shot DSC-F717; Sony Corporation, Tokyo, Japan) by the same operator and under the same conditions (place, light, angle and position of mice) to facilitate reproducibility. Each photograph was classified in accordance with the scores proposed by Takakura et al. [25]: 0 = healthy mucosa; 1 = white patches on up to 20% of the tongue; 2 = white patches on 21 to 90% of the tongue; 3 = white patches on over 91% of the tongue; 4 = thick white patches or pseudomembrane on more than 91% of the tongue.

### Sacrifice of animals

The animals were sacrificed 24 hours (day 12) or 7 days (day 18) after the last application of the proposed treatments by intramuscular injection into the femur region, with a lethal dose of ketamine (200 mg/kg). After the sacrifice, the tongue of each animal was surgically removed and standardized longitudinal sections were performed. The samples were then subjected to histological analyses and RT-qPCR.

### Histological analysis

After fixation in 10% formalin, five-micrometer-thick serial sections were cut, mounted on glass slides and stained with periodic acid-Schiff reagent followed by examination under a light microscope at 200 and 400X magnification. It was evaluated 5 glass slides per sample. Histological changes were evaluated according to the intensity of inflammation, determined by the presence of inflammatory cells, alteration of the population of resident cells on the connective tissue, characteristics of the amorphous intercellular substance and intercellular fibrous substance, according to the following scores: 0: inflammation absent; 1: mild inflammation; 2: moderate inflammation; 3: severe inflammation; 4: abscess formation (ISO 7405:1997). The evaluation was performed by a single examiner blinded to each experimental group at the different time intervals assessed.

### Assessment of gene expression of inflammatory cytokines by RT-qPCR

After storage of the samples in Trizol, 0.1 ml of chloroform (Sigma-Aldrich, St. Louis, MO, USA) was added to each sample and after grinding the samples were incubated for 3 minutes at room temperature. The samples were then centrifuged at 12,000 x*ɡ* (Eppendorf microcentrifuge, Model 5415R, Hamburg, Germany) for 15 minutes at 4°C. After centrifugation, the aqueous phase containing RNA was transferred to a new tube, followed by addition of 0.5 mL of isopropanol (Sigma-Aldrich) to promote RNA precipitation. The samples were incubated at room temperature for 10 minutes and centrifuged at 12,000 x*ɡ* for 10 minutes at 4°C. Then 1ml of 75% ethanol (Sigma-Aldrich) was added followed by centrifugation at 7,500 x*ɡ* for 5 minutes at 4°C. The supernatant was discarded before air-drying the RNA pellet for 45 minutes. The RNA was resuspended in 10 μL of ultra-pure water (Invitrogen) and the samples were incubated for 10 minutes at 55°C (Thermomixer Comfort—Eppendorf, Hamburg, Germany). Then the total RNA obtained was purified using the Qiagen Mini-Elute kit in accordance with the manufacturer’s instructions. The RNA quality and quantity was measured using OD 260 nm and the ratio OD260/280, respectively.

For each total RNA sample (0.5μg/μL), the cDNA was synthesized using the High Capacity cDNA Reverse Transcriptase Kit (Applied Biosystems) according to manufacturer’s recommendations. The cDNA was stored at -20°C until completion of the RT-qPCR.

After cDNA synthesis, the effect of aPDT on the expression of pro- and anti-inflammatory cytokines (IL-1β, IL-6 and TNF-α), involved in the response of superficial infections, was evaluated. Specific primers were selected for each target gene (TaqMan Assay—Applied Biosystems): βActin = Mm00607939_S1; TNF = Mm00443260_G1; IL-1β = Mm00434228_M1; IL-6 = Mm00446190_M1.

It was used the TaqMan assay (Applied Biosystems) for the RT-qPCR reaction (14μL), 1 μL of each specific oligonucleotide for each amplified gene and 1 pg of cDNA from the different samples. Amplification assays were performed in the Step One Plus thermal cycler and amplification cycles were analyzed and determined by the Step One Plus software (Applied Biosystems—Foster City, CA, USA). The process of thermal cycling consisted of 2 minutes at 50°C, 2 minutes at 95°C for reverse transcription, and 40 cycles of 15 seconds at 95°C, 30 seconds at 57°C and 60 seconds at 30°C. In all experiments negative controls were included to rule out possible contamination of RNA by genomic DNA. The RT-qPCR conditions were optimized for each target with optimal concentrations of oligonucleotides, absence of dimer formation of oligonucleotides and efficient amplification of target genes and β-actin control genes. Standard curves were carried out by performing a pool of the samples in the dilution of 1:10 [27]. Data related to cytokine expression of the groups receiving any treatment were compared with the negative control group (healthy animals). All RT-qPCR experiments were performed according to the recommendations of the “MIQE Guidelines for qPCR” [28].

### Statistical analysis

*C*. *albicans* counts (cfu/ml) were transformed into base-10 logarithms. As the data were normal and heteroscedastic, the values were analyzed by two-way ANOVA with the treatment performed at different time intervals (24 h and 7 days after treatment) as main effects. There was interaction between the factors, treatment group and time interval, so one-way ANOVA with Welch correction followed by Games-Howell *post-hoc* test for multiple comparisons was performed. The Spearman correlation test (α<0.01) was used to correlate the number of colony forming units (CFU/mL) with the scores assigned to the lesions on the tongues of animals infected with *C*. *albicans*.

Standard photographs were evaluated by two independent observers blinded to the treatment groups and experimental period. These observers were instructed to classify each photograph of the tongue as score 0 (healthy) or scores 1, 2, 3 or 4. The κ test was used to assess the agreement between the observers and the value was considered significant (κ = 0.73, p≤0.0001).

The data obtained from RT-qPCR for TNF-α cytokine and IL-1 β met the assumptions of normality and homogeneity of variances. Thus, the two-way ANOVA followed by Tukey’s test was used to compare the groups according to the different time intervals assessed (p <0.05). For the cytokine IL-6, the data were normal and heteroscedastic. Therefore, the data were submitted to two-way ANOVA test. As there was interaction between the factors, treatment group and time interval, data were submitted to one-way ANOVA test with Welch correction followed by Games-Howell *post-hoc* test for multiple comparisons. The significance level adopted was 5%.

## Results

### Model establishment

**Fig 2:**
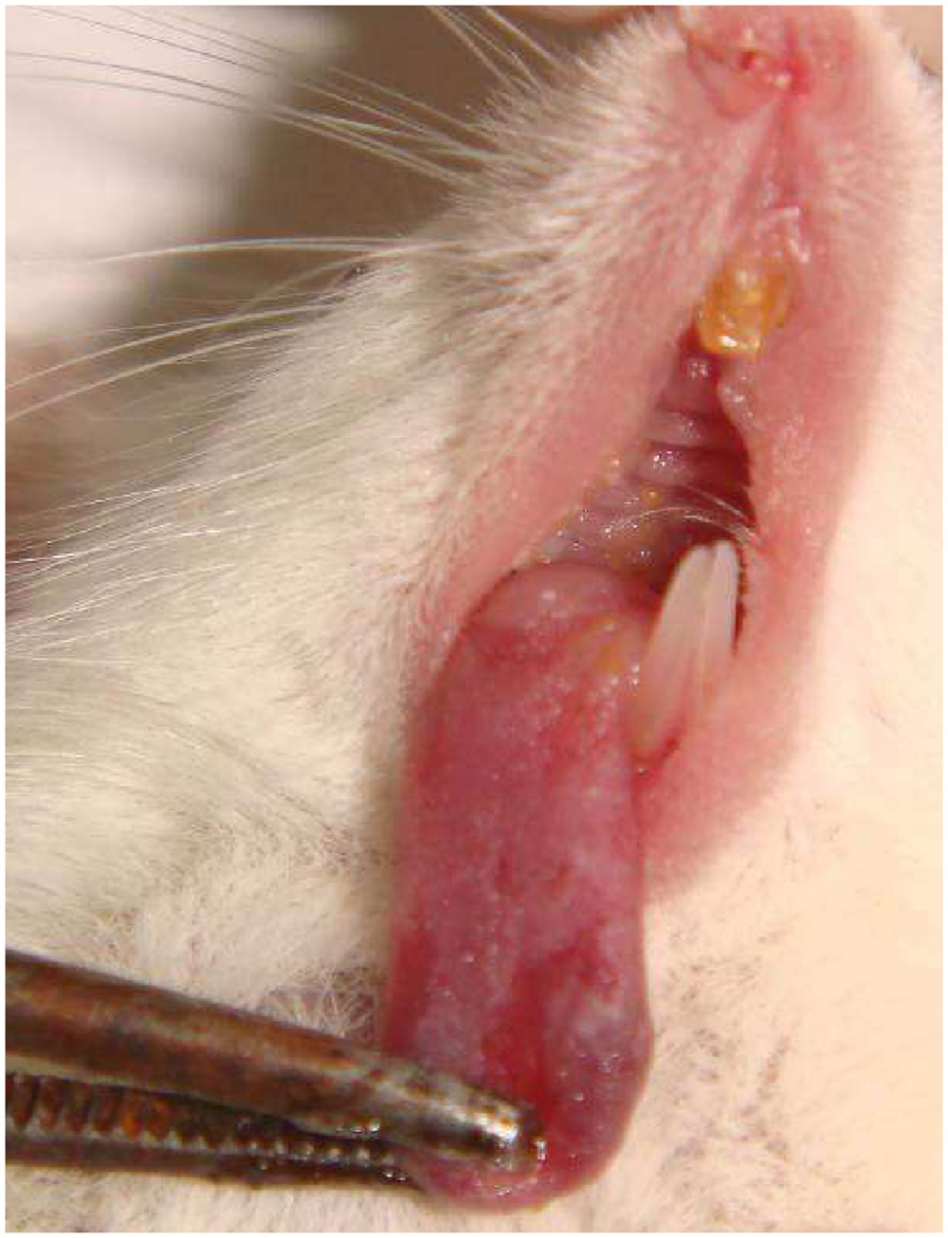
White patches or pseudomembrane present on the dorsum of the tongue of the animals infected with *C*. *albicans* and not treated 5 to 16 days after infection.

**Fig 3:**
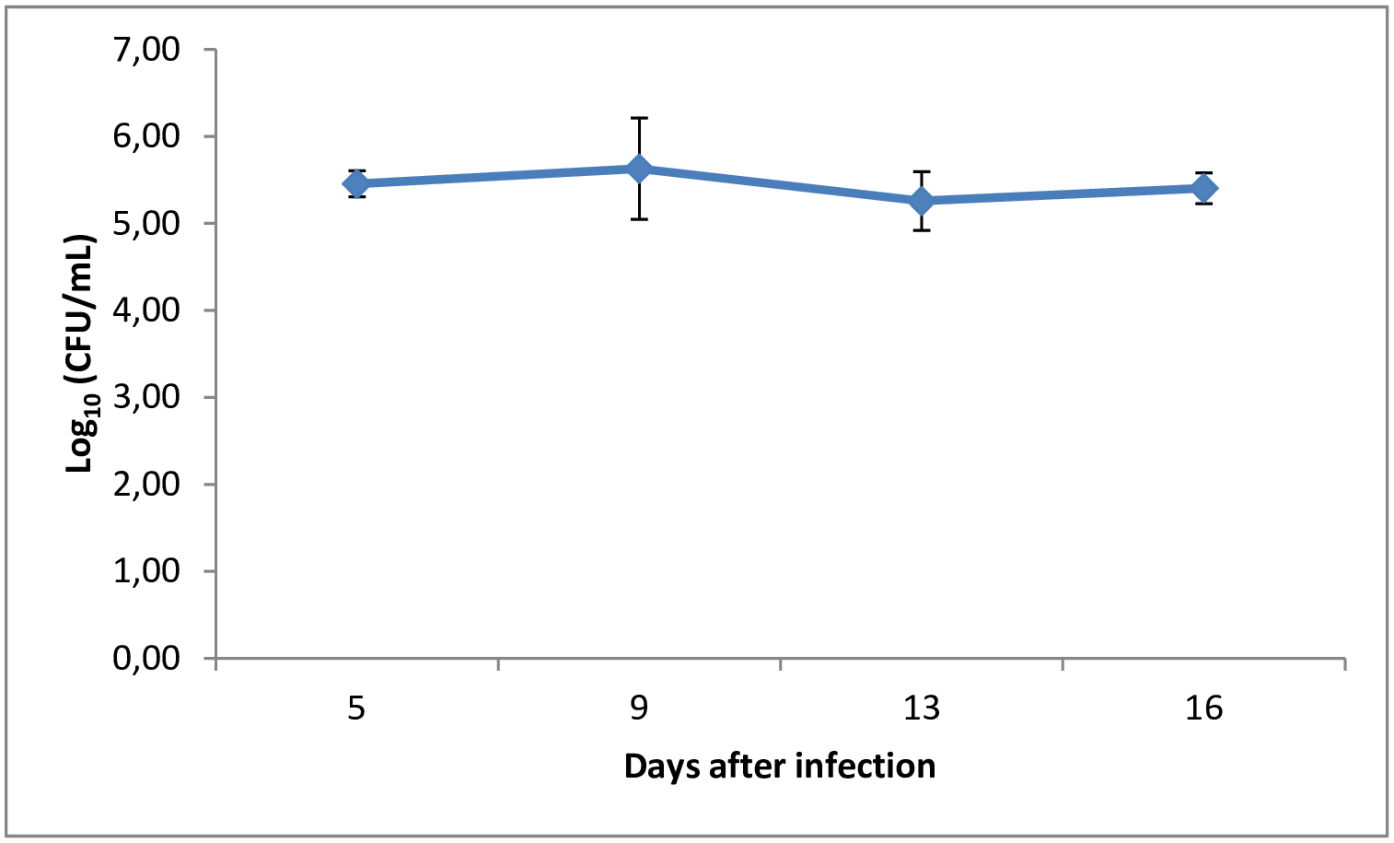
Kinetics of *C*. *albicans* colonization after 5 and 16 days of infection in the established murine model of oral candidiasis.

**Fig 4:**
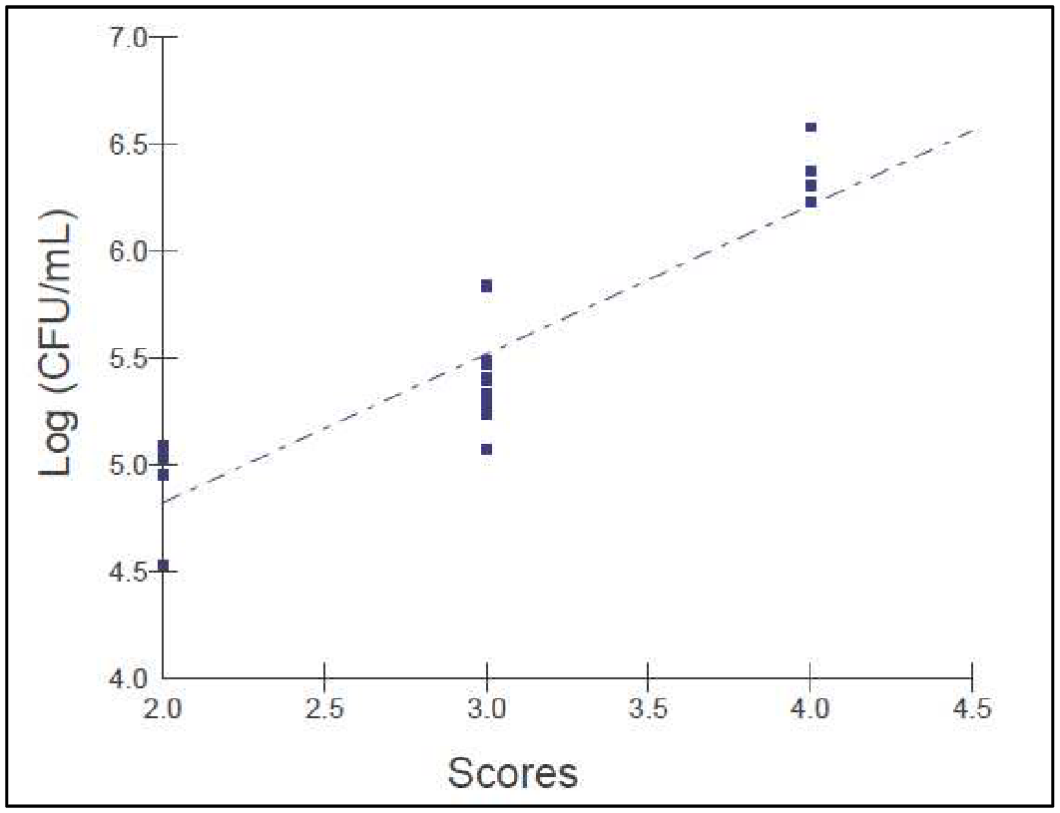
Correlation between the scores assigned to oral lesions with the CFU number/mL within the 5 to 16-day period post-infection in the murine model of oral candidiasis (Rs = 0.92; p≤0.0001).

The murine model of oral candidiasis employed here was successfully established, which could be confirmed by the presence of white patches or pseudomembrane on the tongue of all immunosuppressed animals infected with *C*. *albicans* (score 3) in the control group throughout 16 days (Fig 2). The severity of the lesions was assessed at day 5 until the last day of the experiment and it was recovered 10^5^ CFU/ml of *C*. *albicans* from the oral cavity of animals (Fig 3). Histopathological analysis showed presence of moderate inflammatory infiltrate with extensive colonization of hyphae/pseudohyphae on the entire keratin layer, invading the epithelial tissue of the tongues. The Spearman correlation was performed including all animals infected by *C*. *albicans* during all experimental period. The scores 0 (zero) and 1 were not observed in any mouse. All infected animals presented white patches on at least 25% of the tongue, approximately (score 2). For this reason, the Fig 4 shows the correlation starting in the score 2. Fig 4 shows that a significant positive correlation was found between the scores assigned to oral lesions of the infected animals with the number of CFU/mL within the 5 to 16-day period post-infection (R = 0.92; p≤0.0001).

The behavior of the infected mice was not altered after induction of oral candidiasis. The animals continued feeding and drinking water normally, there was no weight loss or any changes in the amount or volume of urine and feces. It is important to mention that the method developed in the present study did not cause the death of any animal.

### Effectiveness of treatments performed

**Fig 5:**
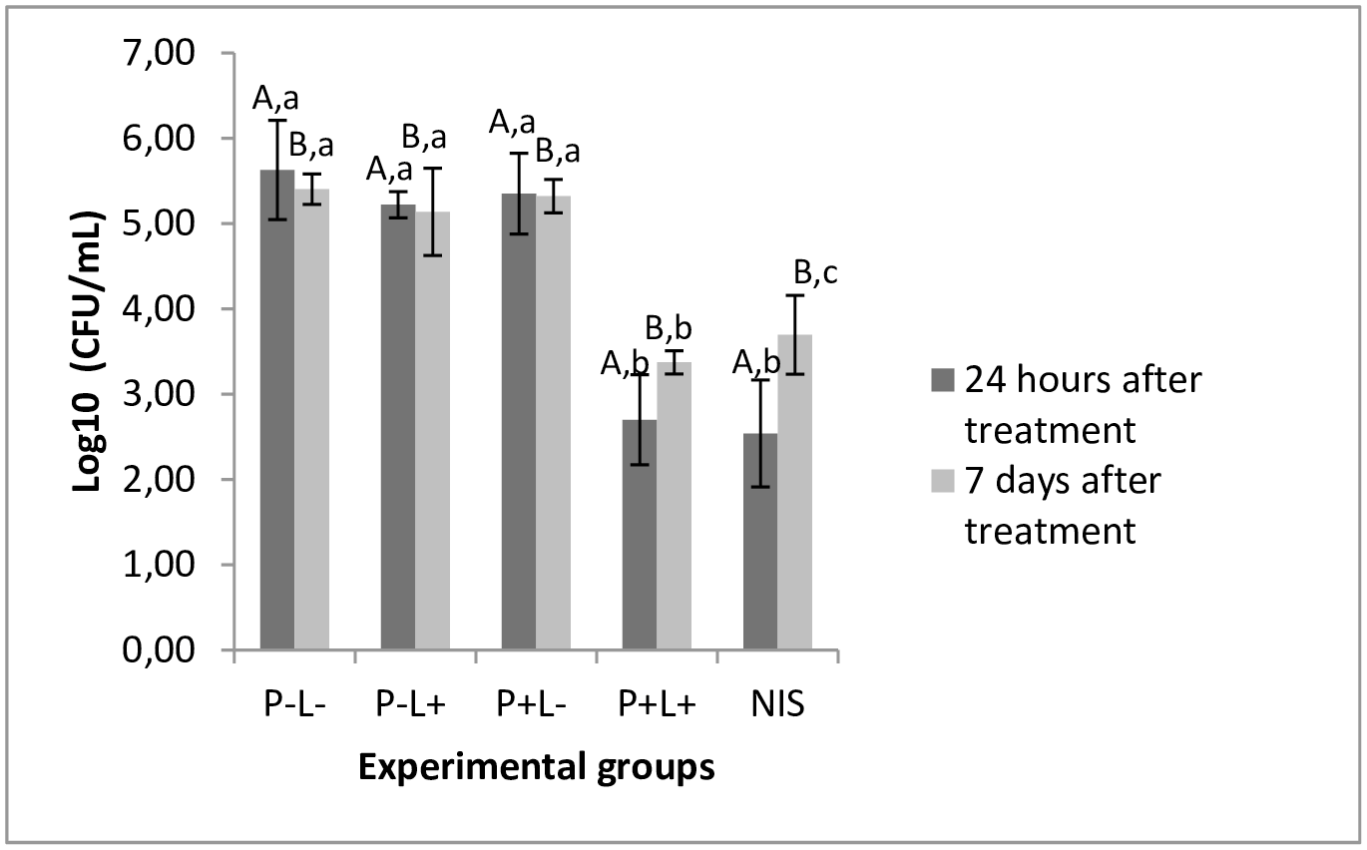
Mean values of log_10_ (CFU/mL) obtained for the control and experimental groups. Different superscript capital letters denote statistical difference between the time intervals assessed (24 hours and 7 days after treatment) and equal superscript lowercase letters denote statistical similarity between the experimental groups (p<0.05). As there was interaction between the factors treatment group and time interval (p≤0.0001) it was performed one-way ANOVA-Welch followed by Games-Howell post-hoc test.

PDZ-mediated aPDT and treatment with NYS promoted significant reduction in yeast viability of approximately 3 and 3.2 log_10_, respectively, when compared to the untreated control group (P-L-) (p≤0.0001 and p≤0.0001 respectively), 24 hours after the treatments. Seven days after treatment, the reduction in log_10_ remained statistically similar to that one observed in the 24-hour time interval in the group treated with aPDT (p≥0.601). There was an increase in the number of CFU/ml for the groups submitted to aPDT and NYS, 7 days after treatment, nevertheless this increase was significant only for the animals treated with NYS (p≤0.001). Moreover, no statistical difference was found among the P-L-, P-L+ and P+L- groups throughout the experimental period (p≥0.05) (Fig 5).

**Fig 6:**
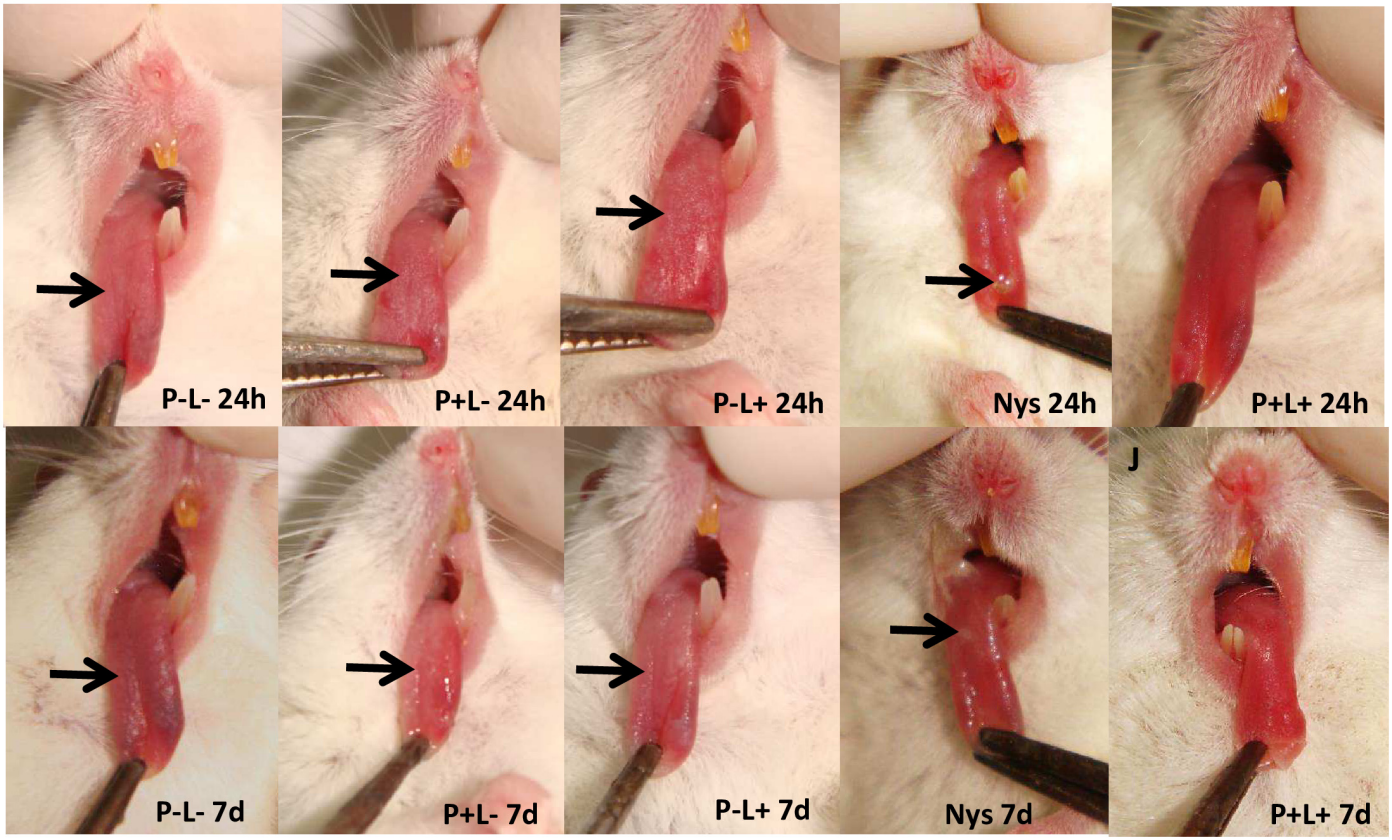
Representative images of white patches or pseudomembrane (arrow) on the dorsum of the tongue of animals from groups P-L-, P+L-, P-L+ and NYS and absence of tongue lesions of the animals submitted to the aPDT (P+L+), 24 hours (24h) and 7 days (7d) after treatment.

**Fig 7:**
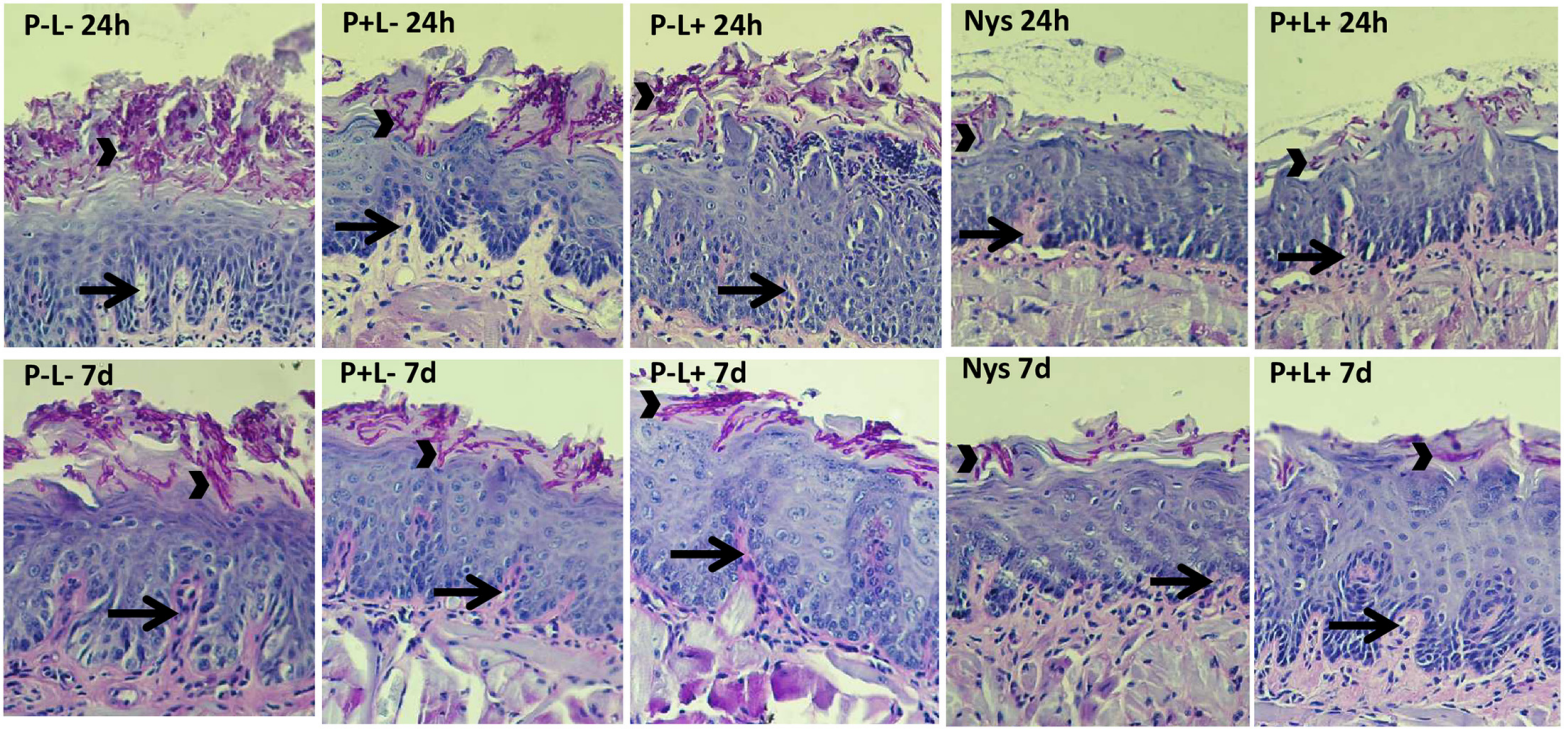
Representative images of histological sections showing a moderate inflammatory infiltrate (arrows) with innumerous hyphae/pseudohyphae (arrow head) on the keratin layer for the groups P-L-, P+L- and P-L+, and a mild inflammatory infiltrate (arrows) with a few hyphae/pseudohyphae (arrow head) on the keratin layer for the groups treated with NYS and P+L+ 24 hours (24h) and 7 days (7d) after treatment.

Twenty-four hours after completion of treatment (5 applications), all animals treated with aPDT presented total remission of lesions (score 0) (Fig 6, P+L+ 24h). For the group treated with NYS, the animals showed partial remission of lesions (score 1) (Fig 6, NYS 24h). The animals treated only with PDZ or light presented white patches on the tongue (score 3) (Fig 6, P+L- 24h and 6P-L+ 24h, respectively). The macroscopic assessment of lesions 7 days after completion of treatment revealed that the scores attributed to lesions remained the same, irrespective of the groups (Fig 6, P-L- 7d, P+L- 7d, P-L+ 7d, NYS 7d and P+L+ 7d).

The histopathological events observed on the tongue tissues of the animals 24 hours after treatments showed the presence of mild inflammatory infiltrates (score 1) for groups treated with aPDT (Fig 7, P+L+ 24h). The group that received NYS presented some hyphae/pseudohyphae on the keratin layer without fungal invasion into the epithelial tissue (Fig 7, NYS 24h). The other groups (Fig 7, P-L+ 24h and P+L- 24h, respectively) presented similar histopathological characteristics observed in the untreated control group (P-L- 24h) with moderate inflammatory infiltration and the presence of numerous hyphae/pseudohyphae on the entire keratin layer, and some hyphae/pseudohyphae invading the epithelial tissue of the tongues (Fig 7, P-L- 24h). The histological findings 7 days after treatments proved to be similar to those observed 24 hours after treatments (Fig 7, P-L-, P+L-, P-L+, NYS and P+L+ 7d).

### Gene expression of TNF-α, IL-1-β and IL-6

**Fig 8:**
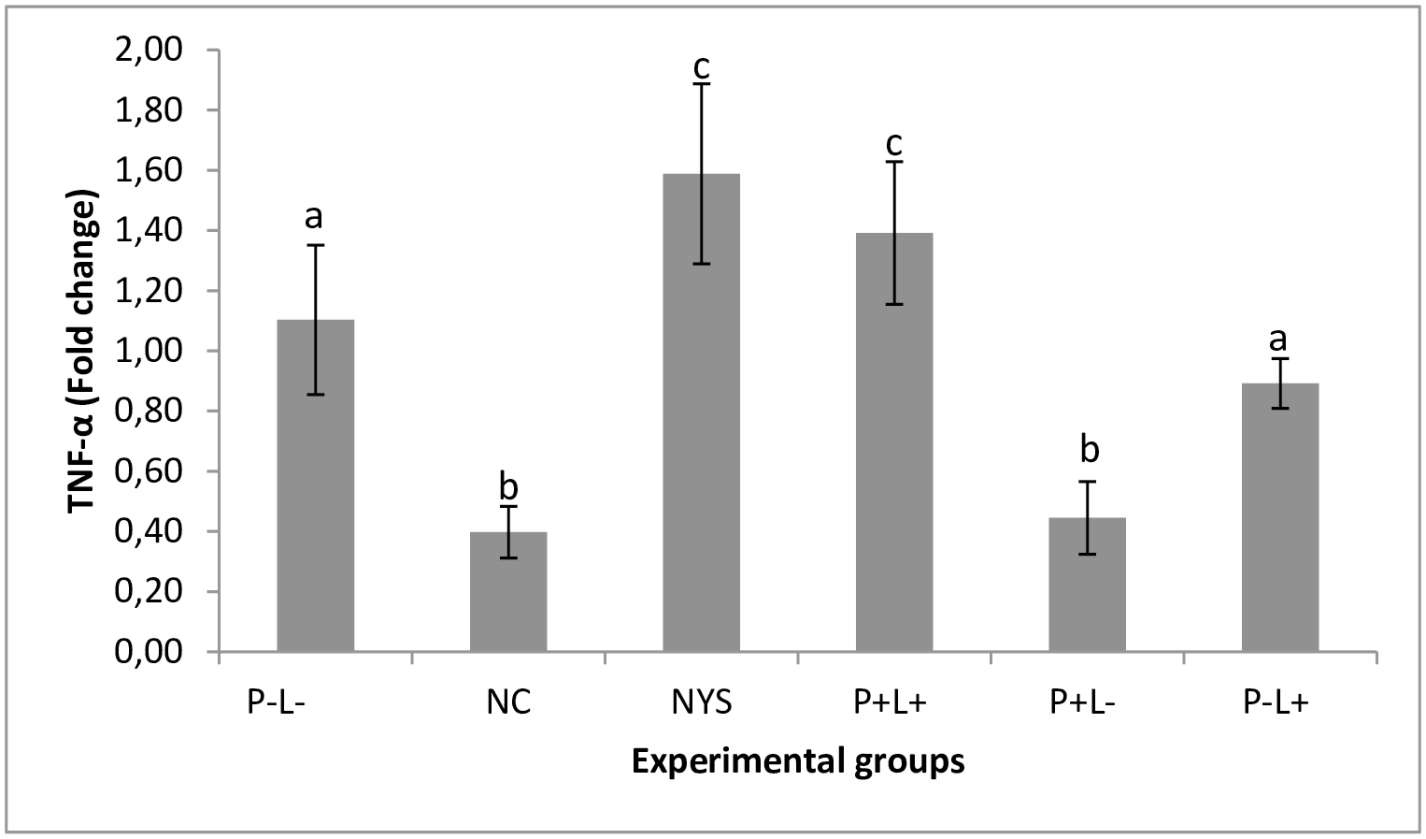
Mean values and standard deviation of the TNF-α gene expression in each experimental group during the time intervals assessed (24 hours and 7 days after treatment). Equal superscript lowercase letters denote statistical similarity among the groups (p>0.05). Data related to cytokine expression of the groups receiving any treatment were compared with the negative control group (NC). It was performed the two-way ANOVA followed by Tukey’s test to compare the groups according to the different time intervals assessed. As there was no interaction between the factors treatment group and time interval (p = 0.986), the mean values of gene expression obtained in the two periods of time were pooled in the same bar.

A significant increase in TNF-α expression was found in the animals submitted to the aPDT (P+L+) (p ≤ 0.014) and NYS (p ≤ 0.007) in comparison with the positive control group. No significant difference was found between the group treated with aPDT and the one treated with NYS (p = 0.605). These results were observed 24 hours and 7 days after treatment. The group treated only with light (P-L+) also showed similar cytokine expression in comparison with the untreated control group (p = 0.530), and the group treated with PDZ showed low cytokine expression similar to the negative control group (p = 0.999) at both time intervals (Fig 8). There was no significant interaction between the experimental groups and period assessed (p = 0.986).

**Fig 9:**
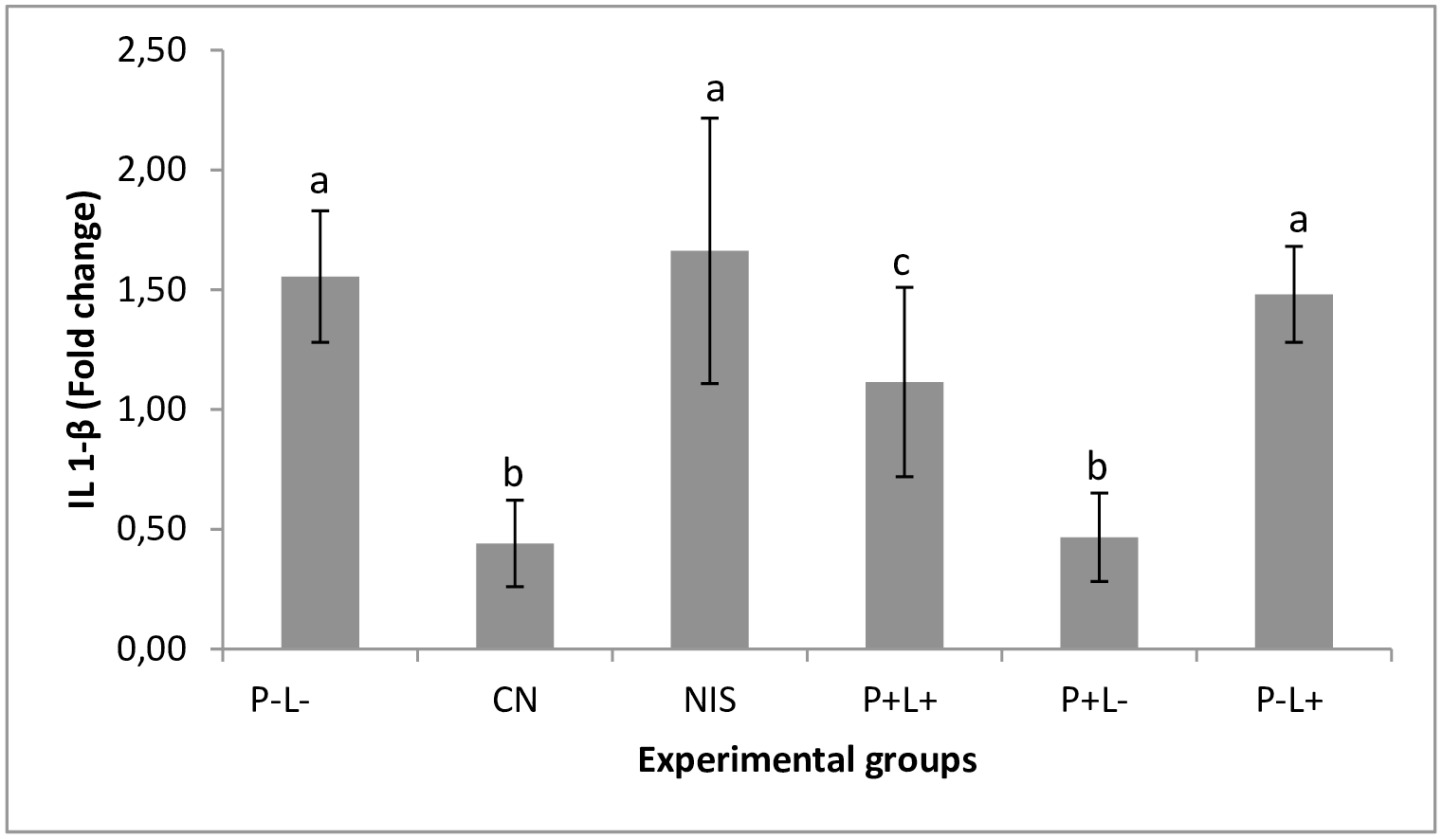
Mean values and standard deviation of the gene expression of IL-1 β assessed for each experimental group depending on the time interval assessed (24 hours and 7 days after treatment). Equal superscript lowercase letters denote statistical similarity between the groups (p>0.05). Data related to cytokine expression of the groups receiving any treatment were compared with the negative control group (healthy animals). It was performed the two-way ANOVA followed by Tukey’s test to compare the groups according to the different time intervals assessed. As there was no interaction (p = 0.105) between the factors treatment group and time interval, the mean values of gene expression were pooled.

The animals submitted to aPDT showed low expression of IL1-β cytokine in comparison with the untreated control group (p = 0.021). The expression of this cytokine in the group of animals treated with NYS was similar to the negative control group (p = 0.979). The group treated only with light (P-L+) also showed cytokine expression similar to the negative control group (p = 0.996). The P+L- group showed low cytokine expression similar to the negative control group (p = 1.000). The above results were observed at both time intervals (Fig 9). No significant difference was found in the interaction between the experimental groups and time interval assessed (p = 0.105).

**Fig 10:**
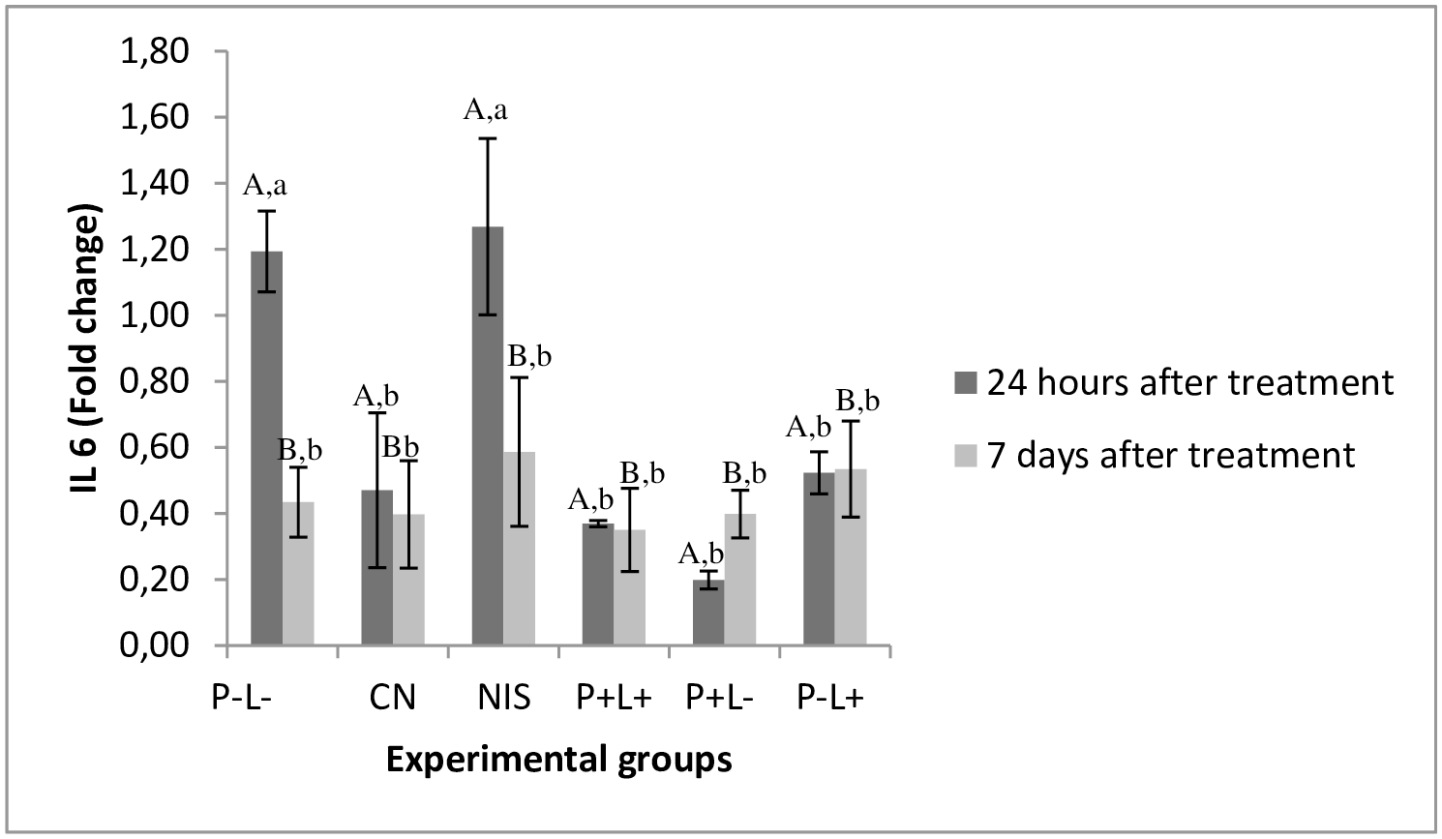
Mean values and standard deviation of IL-6 gene expression evaluated for each experimental group. Data related to cytokine expression of the groups receiving any treatment were compared with the negative control group (healthy animals). Different superscript capital letters denote difference between the time intervals assessed (24 hours and 7 days after treatment) and equal superscript lower case letters denote statistical similarity between the experimental group. As there was interaction between the factors treatment group and time interval (p = 0.048) it was performed one-way ANOVA-Welch followed by Games-Howell post-hoc test.

Regarding the expression of IL-6 cytokine, the group treated with NYS showed similar expression to the untreated control group 24 hours after treatment (p≥0.953). The other groups showed expression similar to the negative control group [P+L+ (p = 0.967), P+L- (p = 0.146) and P-L+ (p = 0.272)]. Seven days after treatment, the cytokine expression in all animals evaluated showed the lowest values similar to those observed in the NC group (p≥ 0.05) (Fig 10). A significant difference was found in the interaction among the experimental groups and time interval assessed (p = 0.048).

## Discussion

The proposed model for the induction of oral candidiasis in immunocompromised mice used in the present investigation is reproducible since all animals infected with *C*. *albicans* showed white patches or pseudomembrane on the dorsum of the tongue within 5 to 16 days after inoculation. In order to allow the monitoring of infection and the establishment of the photodynamic treatment, animals were immunosuppressed 4 times with prednisolone. After fungi inoculation it was collected 10^5^ CFU/ml of *C*. *albicans* from the oral lesions of the animals from the P-L- group (positive control) throughout the experimental period. Histopathological analysis showed presence of moderate inflammatory infiltrate with numerous hyphae/pseudohyphae over the entire keratin layer, and some hyphae/pseudohyphae invading the epithelial tissue of the tongues. These findings are similar to those obtained by Takakura et al., [25] who obtained 10^5^−10^6^ CFU/ml of *C*. *albicans* from the oral lesions of the animals. The histopathological analysis demonstrated the presence of severe inflammatory infiltrate with numerous hyphae, fungal invasion and destruction of epithelial layers. These findings can be explained because Takakura et al. [25] used a clinical strain isolated from patients with cutaneous candidiasis, whereas in the present study it was used a reference strain of *C*. *albicans* for the fungal inoculation. The relation between the scores attributed to the oral lesions and the CFU/mL values was assessed by the Spearman’s correlation coefficient (R = 0.92, n = 12, 5–16 days after infection). This analysis showed a high coefficient value, confirming that the high number of white patches and/or pseudomembrane on the tongue reflected the degree of *C*. *albicans* colonization in the oral cavity of the animals infected. In the study conducted by Takakura et al., [25] the authors also correlated the microbiological data with the degree of oral lesions and observed a high correlation coefficient between these two factors (R = 0.75, n = 26), as shown in this study. The results confirm that the induction of the oral candidiasis model was appropriate for reproducing the infection over an extended period of time, which allows the assessment of the effectiveness of treatments.

Other methodologies have also been proposed to induce oral candidiasis in animals [14, 29]. Teichert et al. [14] used a severe immunodeficiency protocol for inducing oral candidiasis and recovered approximately 2 x 10^2^ CFU/mL of *C*. *albicans*. Totti et al. [28] used sialoadenectomized mice to induce oral candidiasis. The authors performed four inoculations with a suspension of *C*. *albicans*. Among the six animals used for each experimental group, only 2 presented fungal colonization up to 60 days after the infection and only 1 animal up to 75 days after infection. The histopathological analysis revealed the presence of pseudohyphae invading the epithelium, acanthosis and intraepithelial neutrophilic infiltration forming micro-abscesses for the animals that developed oral candidiasis [29]. Although this method facilitated longer colonization of *C*. *albicans*, it was not possible to establish a direct comparison with the present study as the number of CFU/ml from the oral lesions of the animals was not mentioned. Furthermore, in the present investigation, all mice showed typical lesions of oral candidiasis throughout the experimental period. In the study conducted by Totti et al., [29] the infection was not maintained throughout the desired period in most animals.

In the present study, the results showed that PDZ-mediated aPDT was as effective as NYS to inactivate *C*. *albicans* in the oral lesions of mice with experimental oral candidiasis. The association of 100 mg/L of PDZ with 37.5 J/cm^2^ of light promoted a reduction of 3 log_10_ in the cell viability, while NYS showed 3.2 log_10_, when compared with the control group (P-L-), 24 hours after the treatments. There was an increase in the number of CFU/ml for the groups submitted to aPDT and NYS, 7 days after treatments. For the group treated with aPDT the log_10_ of reduction was statistically similar to the reduction achieved 24 hours after therapy, although the reduction observed 7 days after treatment was equivalent to 2.0 log_10_. For the group NYS, the log_10_ of reduction observed was statistically different from that achieved 24 hours after treatment, being equivalent to 1.7 log_10_, suggesting recurrence of infection. These findings are in agreement with the studies that reported frequent recurrence in patients with oral candidiasis weeks after treatment [30–32]. In the present investigation, the antifungal NYS was used as a comparison parameter, as it is considered the most commonly used topical antifungal for the treatment of oral candidiasis [33].

According to the literature, this is the first study that evaluated the effectiveness of successive applications of aPDT for the treatment of oral candidiasis in mice. Thus, it is only possible to make indirect comparisons with the studies reported in the literature as they only used a single application of aPDT. Several photosensitizers have been evaluated for photoinactivation of *C*. *albicans* present in oral lesions in experimental oral candidiasis in mice, such as methylene blue [14], porphyrins [15], erythrosine [34] curcumin [26] and different results have been observed after a single application. Our research group [23] evaluated the effectiveness of PDZ (100 mg/L) associated with LED light (37.5J/cm^2^) *in vivo* and obtained a 4.36 log_10_ reduction in the cell viability after a single application. These findings do not agree with those obtained in the present study because the five successive applications of aPDT performed here, using the same PDZ concentration and light dose of the study conducted by Carmello et al. [23], promoted 3 log_10_ of reduction. In the present investigation, the 5 consecutive applications of aPDT was an endeavor to stablish a treatment for oral candidiasis, aiming the complete inactivation of *C*. *albicans*. It is important to mention that it was achieved an important and significant reduction in the cell viability (3 log_10_), and it was observed a total remission of the oral lesions after treatment. Hypothetically, 5 applications of aPDT should produce more reactive oxygen species (ROS) in cells, which would cause a greater reduction in cell viability when compared with a single application. However, it has been reported that fungal cells have the ability to adapt and protect themselves from oxidative stress and two mechanisms seem to be involved: the first one is related to a decrease in cell membrane permeability of ROS and the second is related to the increase in the regulation of antioxidants and other associated enzymes [35].

On the other hand, macroscopic analysis showed that 24 hours after completion of treatment, all animals submitted to aPDT presented total remission of lesions (score 0). In the group treated with NYS, the animals showed partial remission of lesions (score 1) and the other groups showed extensive lesions on the entire dorsum of the tongue (score 3). These results were maintained up to 7 days after finishing the treatments. In the present study, aPDT was also more effective than NYS in the remission of lesion 24 hours and 7 days after treatment. Two possible hypotheses may explain these findings: the first one may be related to the virulence factor, capacity of adhesion of *Candida albicans* to buccal epithelial cells (BEC) of animals. It has been reported that the capacity of adhesion of *Candida* spp. to BEC is considered a prerequisite for colonization and subsequent successful infection [36]. Soares et al. [36] showed that aPDT mediated by toluidine blue was able to inhibit the growth and adhesion of different clinical isolates of *Candida* spp. to BEC *in vitro*. Regarding the antifungal NYS, one study evaluated the adhesion of *Candida albicans* to BEC of healthy individuals and patients with diabetes mellitus. The authors observed that the antifungal NYS was not able to reduce the adhesion of fungal cells to BEC for diabetic and healthy patients (control) [37]. Thus, it is possible to suggest that aPDT may have reduced the capacity of adhesion of fungal cells to the tongue of mice, which may not have occurred to those treated with NYS. These findings may explain the complete remission of oral lesions from animals treated with aPDT, and partial remission from those treated with NYS. The second hypothesis may be attributed to the biostimulator effect of aPDT. It has been reported that the red LED light is absorbed by components of the cellular mitochondrial respiratory chain, resulting in increased of ROS and adenosine triphosphate (ATP) or cyclic AMP, initiating a signaling cascade that promotes cell proliferation and cytoprotection [38–39]. More specifically, this type of light acts on the cells through the primary photoaceptor: cytochrome c oxidase, the terminal enzyme of the mitochondrial electron transport chain [40–41]. The absorption of light by the cytochrome c oxidase promotes increased amount of ATP and ROS, which leads to increased energy availability and signal transduction [42–43]. These biochemical changes have macroscopic effects, such as increased cell proliferation and consequently the rapid remission of oral lesions. Thus, it seems that PDZ associated with red LED light may have, beyond the fungal inactivation, increased the amount of ROS inside the cell, which stimulated cell proliferation on the tongue tissue of animals, culminating in the complete remission of the oral lesions. This result did not occur in animals treated with NYS and red LED light alone, probably due to the persistence of yeast and fungal infection in the oral cavity. It is also important to mention that the pharmacodynamics and pharmacokinetic effects of NYS in a murine model of oral candidiasis are not completely elucidated in the literature.

Histological analysis of the tongues removed 24 hours after the treatment revealed the presence of mild inflammatory infiltrates (score 1) in the group treated with aPDT. The group that received NYS presented some hyphae/pseudohyphae on the keratin layer, without fungal invasion in the epithelial tissue. The other groups (P-L+, P+L-) were similar to the positive control group (P-L-), which showed moderate inflammatory infiltrate with the presence of numerous hyphae/pseudohyphae over the entire keratin layer, and some hyphae/pseudohyphae invading the epithelial tissue of the tongues. Histological changes found in this study are characteristic of oral candidiasis and have also been reported by other authors [13–15, 34]. With the results of this study, we suggest that the inflammatory infiltrate found in the treated groups is associated with infection caused by *C*. *albicans*. Moreover, histological findings verified 7 days after the treatments showed to be similar to those obtained 24 hours after treatments.

It has been reported that the earliest stages of *Candida* infection induces the local production of TNF-α [44–46]. Here, the animals submitted to aPDT presented TNF-α expression similar to the NYS group and 26% greater than the P-L- group. These results were observed 24 hours and 7 days after finishing the treatments. These findings are in agreement with others studies that suggested the effectiveness of aPDT may be related to TNF-α stimulation [47–50]. According to those authors, a slight increase in TNF-α expression can be considered beneficial since the fungal cells may have been killed or removed by cells during the inflammatory reaction, promoting the rapid resolution of oral lesions.

In addition, we evaluated the expression of IL-1β and IL-6. The results showed that IL-1β expression in the group treated with aPDT was lower than in the P-L- group and the group treated with NYS. For IL-6, the group treated with aPDT showed baseline values of this cytokine, similar to those found in the NC group. The antifungal NYS showed values similar to P-L- group 24 hours after treatments. It is important to point out that these cytokines have a peak release at different times, which may occur within 6 to 8 hours after procedures that stimulate inflammatory reactions [51]. In this study, as the animals were sacrificed 24 hours and 7 days after treatment, it is probably that the expression of these cytokines would have reached a plateau in approximately 8 hours and then decreased, so the difference in expression could not be detected after aPDT treatment. With regard to NYS treatment, the expression of IL-1β and IL-6 was higher than those observed for the NC and aPDT group. It has been reported that NYS is able to elevate the expression of cytokines, such as IL-1β and IL-6 in a murine model with stable dendritic cells [52]. The immunomodulatory effect of NYS may justify the results obtained in the present investigation.

In summary, the oral candidiasis model established in this study was suitable to reproduce the infection. Five successive applications of PDZ-mediated aPDT were as effective as NYS to treat oral candidiasis in immunosuppressed mice. Although histological analysis has shown that the aPDT did not cause adverse effects on the tongue tissue, the biomolecular analysis revealed that this treatment promoted TNF-α expression, which seems to be a beneficial factor for the remission of oral lesions. However, more detailed biomolecular techniques are required to evaluate the production of other pro- and anti-inflammatory cytokines at different time intervals to further explain the mechanism of action of aPDT in the host. Considering the results presented and the fact that the aPDT has been suggested as a therapeutic option for superficial fungal infections, our investigation suggest that aPDT is safe to treat oral candidiasis, since it has antimicrobial activity without harming the host tissue. In the future, these parameters will assist the safe and effective conduction of randomized clinical trials.
